# Isolation of Salt Stress-Related Genes from *Aspergillus glaucus* CCHA by Random Overexpression in *Escherichia coli*

**DOI:** 10.1155/2014/620959

**Published:** 2014-10-14

**Authors:** Jie Fang, Xiaojiao Han, Lihua Xie, Mingying Liu, Guirong Qiao, Jing Jiang, Renying Zhuo

**Affiliations:** ^1^State Key Laboratory of Tree Genetics and Breeding, Chinese Academy of Forestry, Beijing 100091, China; ^2^Key Lab of Tree Genomics, The Research Institute of Subtropical of Forestry, Chinese Academy of Forestry, Fuyang, Zhejiang 311400, China

## Abstract

The halotolerant fungus *Aspergillus glaucus* CCHA was isolated from the surface of wild vegetation around a saltern with the salinity range being 0–31%. Here, a full-length cDNA library of *A. glaucus* under salt stress was constructed to identify genes related to salt tolerance, and one hundred clones were randomly selected for sequencing and bioinformatics analysis. Among these, 82 putative sequences were functionally annotated as being involved in signal transduction, osmolyte synthesis and transport, or regulation of transcription. Subsequently, the cDNA library was transformed into *E. coli* cells to screen for putative salt stress-related clones. Five putative positive clones were obtained from *E. coli* cells grown on LB agar containing 1 M NaCl, on which they showed rapid growth compared to the empty vector control line. Analysis of transgenic *Arabidopsis thaliana* lines overexpressing *CCHA-2142* demonstrated that the gene conferred increased salt tolerance to plants as well by protecting the cellular membranes, suppressing the inhibition of chlorophyll biosynthesis. These results highlight the utility of this *A. glaucus* cDNA library as a tool for isolating and characterizing genes related to salt tolerance. Furthermore, the identified genes can be used for the study of the underlying biology of halotolerance.

## 1. Introduction

Halotolerant or halophilic microorganisms, able to live in saline environments, offer a large number of potential applications in various fields of biotechnology [1]. In order to survive high salinity, such organisms have developed adaptive features to function under extreme conditions [2]. Salinity stress is one of the major factors that reduce crop plant growth and productivity, resulting in crucial economic losses worldwide. Therefore, it is vital to isolate and functionally characterize salinity stress-related genes to elucidate the mechanisms underlying halotolerance and develop salinity stress-tolerant plants [3].

As the fundamental cellular adaptive mechanisms are conserved and play precise roles in salinity stress [4], it can be supposed that stress-related genes from various species can be screened efficiently via overexpression in simple organisms. Indeed, there are several reports of such functional screening of stress-related genes by overexpression in yeast [5–7] or* E. coli *[8–10]. The gene for a catalytic subunit of the* Beta vulgaris* (sugar beet) protein kinase CK2 (*BvCKA2*) related to salinity was cloned by functional expression in yeast of a NaCl-induced* B. vulgaris* cDNA library [6]. In addition, a plant RelA/SpoT homolog has been found to confer salt tolerance to* E. coli* [10]. It is thought that the cellular response to environmental stress is highly conserved among all three superkingdoms, namely, the eukaryotes, the eubacteria, and the archaea [4]. Although plants may have unique adaptive mechanisms to accommodate various stresses, the basic cellular stress response (CSR) mechanism, which is activated in response to stress, is similar in prokaryotes, lower plants, and eukaryotes [4, 10]. Accordingly, stress-resistance genes of halotolerant fungal species have the potential to improve the stress tolerance of microorganisms and plants [11–14].

The* Aspergillus glaucus* (*A. glaucus*) CCHA strain is one of the salt-tolerant fungi that are found to thrive in salt mines. Its optimum salt concentration is 50 g L^−1^ and it can survive from 0 g L^−1^ to 310 g L^−1^ salt [15]. Therefore,* A. glaucus* might serve as a valuable resource from which salt tolerance related genes are identified. The isolation and identification of salinity stress-resistance genes will promote effort to develop plants tolerant of salinity stress. In this study, an* A. glaucus* CCHA cDNA library was constructed and functionally screened for halotolerance-related genes. A number of clones were identified, and five positive clones were analyzed for their roles in stress tolerance. The results demonstrate that this cDNA library represents an efficient tool for isolating and characterizing genes related to salt tolerance.

## 2. Materials and Methods

### 2.1. Media, Strains, and Growth Conditions

*A. glaucus* (strain CCHA), which was isolated and characterized at Jilin University, China [15], was kindly provided by Professor Shihong Zhang. It was cultivated using potato dextrose agar (PDA) and potato dextrose broth (PDB) with 0.85 M NaCl. Liquid cultures were grown at 35°C on a rotary shaker at 180 rpm.

*E. coli* DH5*α* and BL21 Star (DE3) cells were grown at 37°C on Luria-Bertani (LB) medium with the addition of antibiotics in accordance with the requirements of the vectors. Liquid cultures were grown at 37°C on a rotary shaker at 200 rpm.

### 2.2. RNA Isolation

For RNA isolation,* A. glaucus* (strain CCHA) was grown in PDB liquid medium with 3 M NaCl and harvested by centrifugation after 4 days. The pellet was frozen in liquid nitrogen and homogenized using a mortar and pestle. Total RNA was isolated from 200 mg homogenized tissue using a Norgen RNA Purification Kit (Norgen Biotek Corp., Ontario, Canada). The quality and quantity of total RNA were analyzed using a NanoDrop 2000 UV-Vis spectrophotometer (Thermo, Wilmington, USA) and gel electrophoresis.

### 2.3. Library Construction in* E. coli* DH5*α*

A cDNA library was constructed using the SMART cDNA Library Construction Kit (Clontech, Mountain View, USA) according to the manufacturer's instructions with minor modifications. Briefly, the first strand cDNA was synthesized from 5 *μ*g total RNA using SuperScript III (Clontech, Mountain View, USA) with SMART IV oligonucleotide primer and CDS III/3′ PCR primer (Table 1). The products were used for the synthesis of second strand cDNA catalyzed by 50× Advantage 2 Polymerase Mix. The resulting double strand cDNA (ds cDNA) (2-3 *μ*g in 50 *μ*L) was digested by proteinase K (20 *μ*g/*μ*L) to inactivate the DNA polymerase. The purified ds cDNA was digested with* Sfi*I at 50°C for 4 h, 2 *μ*L 1% xylene cyanol dye was added, and cDNA size fractionation was performed using CHROMA SPIN-400 (Clontech, Mountain View, USA). The concentration and yield of the ds cDNA fractions were estimated using a NanoDrop 2000 UV-Vis spectrophotometer (Thermo, Wilmington, USA) and gel electrophoresis. The ds cDNA longer than 700 bp was pooled, precipitated, and used for ligation reactions with modified vector pYES2G digested by* Sfi*I [16]. Subsequently, 5 *μ*L products of the reaction were electroporated into 100 *μ*L* E. coli* Electro-Cells DH5*α* (TAKARA, Dalian, China), at 1500 V with a Gene Pulser (BIO-RAD, Hercules, USA). The titer of the library was determined by serial dilution in LB medium (0, 10^−1^, 10^−2^, and 10^−3^). In order to estimate the integrity and annotation of the inserts, 100 clones were sequenced randomly. The library was mixed with the freezing medium (15% glycerol, 85% LB medium), frozen, and stored at −80°C.

### 2.4. Library Transfer to* E. coli* BL21 Star (DE3)

An aliquot of the library that contained approximately 4.8 × 10^5^ transformants was inoculated into 50 mL liquid LB medium with ampicillin and grown on a rotary shaker at 180 rpm and 37°C. After overnight incubation, total plasmid DNA was isolated with the AxyPrep Plasmid Miniprep Kit (Axygen Scientific, Tewksbury, USA). The plasmid DNA was transformed into* E. coli* BL21 Star (DE3) cells by electroporation.

### 2.5. Identification of Clones Conferring Salt Tolerance in* E. coli* BL21 Star (DE3)

To characterize the growth of transformants under salt stress, 100 *μ*L* E. coli* BL21 Star (DE3) library aliquots were plated on LB plates (100 mg L^−1^ ampicillin) containing a range of concentrations of NaCl (0.7, 0.75, and 0.85 and 1 M). The screening plates were then incubated at 37°C for 48 h and transformants with increased salt tolerance were selected for further study. PCR was performed to assess the insert in the pYES2G vector in a 20 *μ*L reaction volume, with 10 p mol vector-specific primers (T7 and pYES2G-R). The thermal profile of the reaction was as follows: 5 min denaturation at 95°C, followed by 35 cycles of 30 s at 95°C, 30 s at 53°C, and 2 min and 30 s at 72°C, with the final elongation step of 7 min at 72°C. The PCR products were analyzed by agarose electrophoresis and the colonies with the desired length were sequenced by Sangon Biotech Co., Ltd. (Sangon, Shanghai, China). The nucleotide sequences were analyzed and their putative functions were identified by comparison with GenBank database entries using BLASTX. All colonies showing tolerance to salt stress were mixed with the freezing medium (15% glycerol, 85% LB medium), frozen, and stored at −80°C until further analysis.

### 2.6. Salt-Tolerance Assays

Salt tolerance of the positive* E. coli* BL21 Star (DE3) transformants was compared to that of transformants carrying the empty vector. Spot assays were carried out to ascertain the functions of the screened genes from* A. glaucus* (strain CCHA) in* E. coli* BL21 Star (DE3) cells. Cells were grown in LB medium until OD 600 reached 0.6. Then, the cultures were serially diluted to 10^−3^, 10^−4^, and 10^−5^, and 5 *μ*L of each dilution was spotted on LB plates supplemented with 0.7 M NaCl or 1 M NaCl and cultured for 3 d.

Functional analyses of five clones showing tolerance to 1 M NaCl were also performed in LB liquid culture supplemented with 1 M NaCl.* E. coli* BL21 Star (DE3) cells with recombinant plasmid or empty vector were grown in LB liquid culture, diluted to OD 600 = 0.6, and 1 mL cells were inoculated in 100 mL LB medium containing 1 M NaCl and incubated at 37°C, 200 rpm. The bacterial suspensions were harvested every 2 h through 14 h and OD 600 was measured.

### 2.7. Real-Time Quantitative PCR Analysis

*A. glaucus* samples were grown in PDB medium with 1 M NaCl at 35°C and 180 rpm. After 2 d, mycelium pellets were harvested and inoculated in PDB medium with 3 M NaCl for 0 (control), 0.5, 1, 6, 24, 48, and 72 h. Mycelium pellets were harvested and used for the extraction of RNA. The cDNA was prepared using 1 *μ*g total RNA with the PrimeScript RT reagent kit (TAKARA, Dalian, China). The expression profiles of five representative genes,* CCHA-2142*,* CCHA-2114*,* CCHA-2227*,* CCHA-2229*, and* CCHA-2247*, in response to salt stress were determined with tubulin as the internal reference. The primers were designed using primer 5 (Table 1). The reaction mixture in a total reaction volume of 20 *μ*L included 2 *μ*L cDNA, 10 *μ*L SYBR Premix Ex Tag (2×) (TAKARA, Dalian, China), and 0.4 *μ*L gene-specific primers. The cycling conditions were as recommended by the manufacturer (10 s at 95°C, 40 cycles of 95°C for 5 s, and 60°C for 31 s). Specificity of amplification was verified by melting curve analysis (60 to 95°C) after 40 PCR cycles. The experiments were repeated three times independently. The relative expression was calculated according to the method of Livak and Schmittgen [17].

### 2.8. Verifying the Salt Stress Tolerance Activity of Genes in* Arabidopsis thaliana*

*CCHA-2142* cDNA was digested with* Sfi*I and then inserted into the pBI121G vector, which is remodeled as previously described [18]. The recombinant vector was transformed into* Agrobacterium tumefaciens* EHA105 by electroporation at 2500 V.* Arabidopsis thaliana* ecotype Columbia plants were transformed by the floral dip method [19]. T_0_ seeds were collected and sown on sterile medium containing 50 *μ*g mL^−1^ kanamycin and then confirmed by PCR and RT-PCR using the primers described above and those for* Actin2* (Table 1) as the internal control. Wild-type plants (ecotype Columbia) and T_3_ *CCHA-2142*-overexpressing* Arabidopsis thaliana* plants were sown in soil under a long-day cycle (16 h light, 8 h dark). After 30 d, transgenic and control wild-type plants were soaked in Hoagland's medium with, for 1 d, 3 d, and 5 d, or without 200 mM NaCl. Then 0.5 g leaf tissue was ground in liquid nitrogen and homogenized in 10 volumes of extraction buffer (45.8 mM Na_2_HPO_4_ *·*12H_2_O, 4.25 mM NaH_2_PO_4_ *·*2H_2_O, pH 7.8) per unit fresh weight. After centrifugation at 12000 ×g for 30 min at 4°C, the supernatant was used for measurements of total superoxide dismutase (SOD) activity and malondialdehyde (MDA) content.

Total SOD activity was assayed by the method described in Giannopolitis and Ries [20]. MDA, a biomarker for lipid peroxidation, was assayed by the method described in Heath and Packer [21]. Leaf tissue (0.2 g) and 20 mL ultrapure water were mixed, and 3 hours later, the electrical conductivity was measured. Then the mixtures were heated in boiling water for 20 min and then cooled to 25°C to determine the total electrical conductivity. The degree of ion leakage was calculated as electrical conductivity (before boiling)/electrical conductivity (after boiling) *·* 100%. Leaf tissue (0.1 g) was used for chlorophyll content measurements. Total chlorophyll content, chlorophyll a content, and chlorophyll b content were measured after extraction with 80% acetone [22]. All the experiments were carried out independently at least thrice with four lines (wild type and 3 transgenic plant lines).

## 3. Results

### 3.1. Identification of* A. glaucus* Genes Expressed in the Presence of Salt

We began by constructing a cDNA library from* A. glaucus* (strain CCHA) grown on medium containing 3 M NaCl. To gain an overview of genes expressed in* A. glaucus*, one hundred clones were randomly selected for sequencing and bioinformatics analysis. The sizes of the inserted cDNA fragments in the library were ~500–1500 bp. The sequences were annotated based on alignment with sequences in various databases including the National Center for Biotechnology Information (NCBI) nonredundant protein (Nr) database, the NCBI nonredundant nucleotide sequence (Nt) database, and UniProt/Swiss-Prot. Of the 100 unigenes analyzed, 71 had significant matches in the Nr database, Nt database, and Swiss-Prot database. Based on their similarity to known proteins, the annotated genes appear to be functionally involved in signal transduction, osmolyte synthesis and transport, and regulation of transcription (Table 2).

### 3.2. Screening for* A. glaucus* Genes Conferring Salt Tolerance

The* A. glaucus* cDNA library was then transferred to* E. coli* BL 21 Star (DE3) cells for expression. We performed a salt screen assay, in which the* E. coli* BL21 Star (DE3) library was plated on LB containing 0.7, 0.75, and 0.85 M NaCl. Whereas there was a lawn of colonies on LB plates (containing 0.7 M NaCl), many fewer colonies survived on LB plates containing higher concentrations of NaCl. Sixty transformants that could grow on LB with 0.75 M NaCl were sequenced, and the sizes of the inserts ranged from 300 to 1,500 bp. In addition, a total of 22 unique sequences were identified based on supporting growth on LB containing 0.85 M NaCl. Putative functions were identified for 20 of the genes by comparison with deposited sequences in the GenBank database (Table 3). Notably,* A. glaucus* CCHA-1408 was most similar to RNase PH-related exoribonuclease, which appears to be an important salt stress protein [23]. Two other genes,* CCHA-2227* and* CCHA-2114*, belonged to the DJ-1/Pfpl family, which may be related to salt stress [24]. Based on their putative functional annotations, 14 transformants were selected for further analyses.

### 3.3. Analysis of Salt Tolerance Conferred by Selected Clones from* A. glaucus* in* E. coli* BL21 Star (DE3)

**Figure 1:**
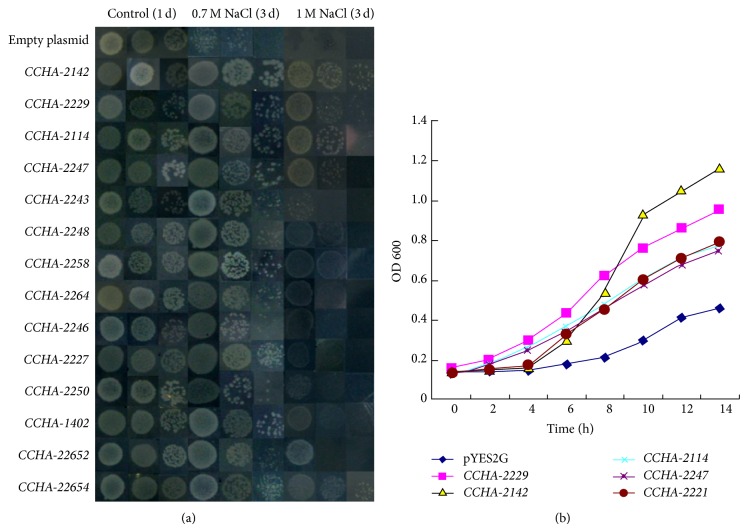
*A. glaucus* genes confer salt tolerance to* E. coli*. (a) Spot assay of BL21/pYES2G (empty plasmid), BL21/CCHA-2142, BL21/CCHA-2229, BL21/CCHA-2114, BL21/CCHA-2247, BL21/CCHA-2243, BL21/CCHA-2248, BL21/CCHA-2258, BL21/CCHA-2264, BL21/CCHA-2246, BL21/CCHA-2227, BL21/CCHA-2250, BL21/CCHA-1402, BL21/CCHA-22652, and BL21/CCHA-22654 on LB plates with 0.7 M NaCl and 1 M NaCl. (b) Growth analysis of* CCHA-2142*,* CCHA-2114*,* CCHA-2229*,* CCHA-2227*, and* CCHA-2247* in LB liquid medium with 1 M NaCl. OD_600_ was recorded every 2 h.

Spotting assays of selected transformants on LB plates (Figure 1(a)) did not reveal any differences in growth at 0.5 M NaCl. By contrast, while the control strain carrying an empty plasmid did not grow on LB containing 0.7 or 1 M NaCl, all of the selected* A. glaucus* clone transformants grew at 0.7 M NaCl. Five of them (*CCHA-2142*,* CCHA-2114*,* CCHA-2221*,* CCHA-2229*, and* CCHA-2247*) could support growth at 1 M NaCl, and their accession numbers are KJ934998, KJ935001, KJ935002, KJ934999, and KJ935000, respectively (Table 4). The growth of these five transformants was also analyzed in LB liquid medium containing 1 M NaCl. All five showed increased growth compared to the control strain with empty vector alone (Figure 1(b)).

### 3.4. Bioinformatics Analysis of* A. glaucus CCHA-2247*,* CCHA-2229*, and* CCHA-2142*

The three genes conferring the strongest resistance to salt were chosen for detailed bioinformatics analysis. The* CCHA-2247* cDNA contains a predicted gene with a full-length coding sequence of 408 bp encoding a 135 aa protein of 15.6 kDa. The predicted pI is 10.53 and there is no signal peptide. According to the CELLO program (http://cello.life.nctu.edu.tw/), this protein may be located in the periplasm. CCHA-2247 showed 93% identity to ribosomal L27 protein of* Aspergillus flavus* (XP_002381915.1), 93% identity to ribosomal L27 protein of* Aspergillus terreus* (XP_001212185.1), and 92% identity to ribosomal L27 protein of* Aspergillus clavatus* (XP_001269541.1) at the amino acid level. Phylogenetic analysis of CCHA-2247 revealed that it clustered with a ribosomal protein from* Aspergillus flavus* (Figure S1b, see Supplementary Material available online at http://dx.doi.org/10.1155/2014/620959).

The* CCHA-2229* cDNA includes a gene with a predicted full-length coding sequence of 1038 bp. The deduced amino acid sequence revealed a protein consisting of 345 aa with a predicted molecular mass of 37.9 kDa. As predicted, the pI is 5.54 and it has no signal peptide. Using the TMHMM program (http://www.cbs.dtu.dk/services/TMHMM/), we predicted that it is an inner membrane protein with 8 transmembrane domains. The BLASTP analysis results suggested that it is a putative plasma membrane low affinity zinc ion transporter. CCHA-2229 shares 94% identity with proteins from* Aspergillus ruber* (EYE97956.1) and 76% identity with one from* Aspergillus clavatus* (XP_001272799.1). Phylogenetic analysis placed CCHA-2229 at the base of the putative plasma membrane low affinity zinc ion transporter family (Figure S1d), which indicates that it may be an ancient form of a plasma membrane low affinity zinc ion transporter.

The cDNA of* CCHA-2142* encodes a predicted protein consisting of 347 amino acids with a molecular mass of 39.5 kDa. There is a signal peptide in this sequence and its pI is 6.06. Preliminary analysis result of subcellular localization showed that it is likely to be a periplasmic protein. The amino acid sequence alignment of CCHA-2142 is shown in Figure S1e. In addition, sequence analysis showed that this gene belongs to DUF3431 superfamily. CCHA-2142 shares 66% identity with an* Aspergillus terreus* protein (XP_001213981.1), 62% identity with a* Neosartorya fischeri* protein (XP_001262501.1), and 61% identity with an* Aspergillus fumigatus* protein (XP_746495.1). Phylogenetic analysis of CCHA-2142 indicated that it clustered with one hypothetical protein of* Neosartorya fischeri* NRRL (XP_001262501) (Figure S1f).

### 3.5. Expression Profiles of* CCHA-2142*,* CCHA-2114*,* CCHA-2227*,* CCHA-2229*, and* CCHA-2247* in* A. glaucus* under Salt Stress

**Figure 2:**
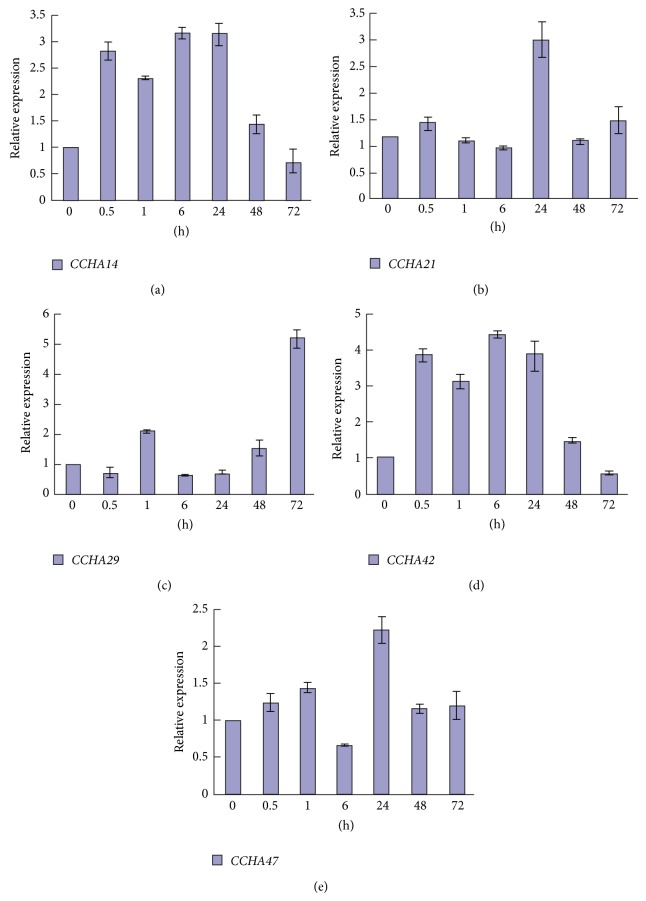
Expression patterns of candidate* A. glaucus* salt-tolerance genes. Real-time PCR analysis of* CCHA-2114* (a),* CCHA-2221* (b),* CCHA-2229* (c),* CCHA-2142* (d), and* CCHA-2247* (e) in* A. glaucus* CCHA under 3 M NaCl stress for 0, 0.5, 1, 6, 24, 48, and 72 h.

Quantitative real-time PCR was performed to analyze the expression patterns of selected candidate halotolerance-conferring genes from* A. glaucus*. Overall, all five genes showed upregulation in response to salt stress, albeit to various degrees. The expression profiles of* CCHA-2114* (Figure 2(a)) and* CCHA-2142* (Figure 2(d)) in* A. glaucus* under salt stress were similar to each other, with an immediate response at 0.5 h, reaching a peak at 6 h or 24 h and then gradually decreasing to the original level at 72 h. The expression of* CCHA-2229* showed no obvious upregulation until treatment had lasted for 72 h, at which point expression was increased 5.17-fold (Figure 2(c)).* CCHA-2221* (Figure 2(b)) and* CCHA-2247* (Figure 2(e)) were upregulated 2.57-fold and 2.22-fold, respectively, at 24 h of salt stress, with no significant change at other treatment durations.

### 3.6. *A. glaucus CCHA-2142* Confers Salt Tolerance to* Arabidopsis thaliana* in Transgenic Plants

**Figure 3:**
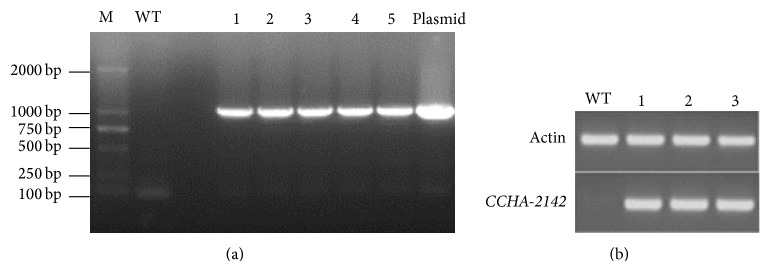
The identification of* CCHA-2142*-overexpressing* Arabidopsis thaliana*. (a) PCR analysis of transgenic plants with* CCHA-2142*: M means DM 2000 marker; WT means wild type; 1, 2, 3, 4, and 5 mean transgenic lines; and plasmid means positive control (plasmid pBI121G-*CCHA-2142*). (b) Confirmation of* CCHA-2142* was expressed in transgenic lines by RT-PCR; WT means wild type; and 1, 2, and 3 mean transgenic lines.

**Figure 4:**
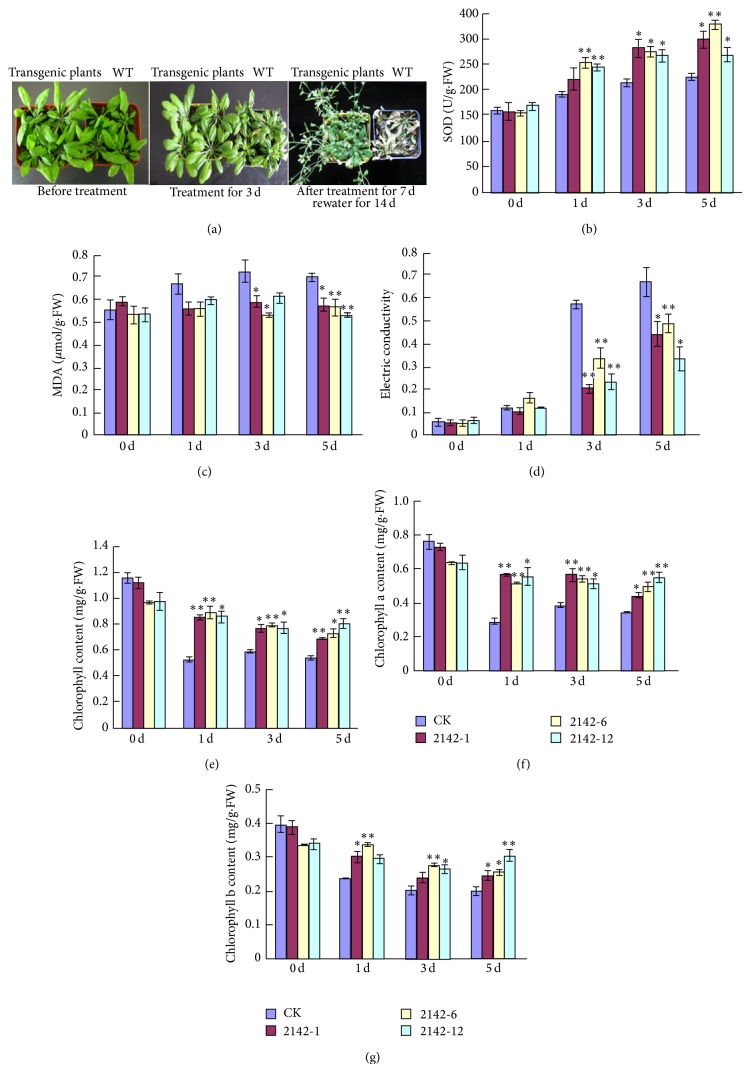
Physiological comparison of control and* CCHA-2142*-overexpressing* Arabidopsis thaliana*. (a) Salt tolerance of transgenic plants and WT. Delayed senescence was observed for the transgenic line as compared with WT upon stress with 200 mM NaCl for 3 d. Comparison of SOD activity (b), malondialdehyde (MDA) content (c) and electrical conductivity (d), total chlorophyll content (e), chlorophyll *a* content (f), and chlorophyll *b* content (g) between* CCHA-2142*-transformed and wild-type* Arabidopsis thaliana* plants under 200 mM NaCl stress for 1 d, 3 d, and 5 d. 0 d: without stress. Lane CK: negative control (wild-type* Arabidopsis thaliana*); Lines 2142-1, 2142-6, and 2142-12: transgenic* Arabidopsis thaliana* lines. Data represent means ± standard deviations (SD) of three biological replicates. One asterisk and two asterisks indicate significant differences at *P* ≤ 0.05 and *P* ≤ 0.01 compared with WT by Student's *t*-test, respectively.

To examine the potential ability of* CCHA-2142* to confer salt tolerance to other species, we generated transgenic* Arabidopsis thaliana* lines constitutively expressing* A. glaucus CCHA-2142* which were detected by PCR (Figure 3(a)) and RT-PCR (Figure 3(b)). Three transgenic lines and a control line (wild type, WT) were used for physiological studies under salt stress. In total, more serious damage was observed on the leaves of the wild-type* Arabidopsis* than that of the transgenic lines when treated with 200 mM NaCl. After 72 hours, both the wild-type and transgenic plants began to wilt, while injurious spots gradually appeared on the leaves of wild-type plants, which was not observed on transgenic lines. And the growth of the wild type was much more inhibited compared to the transgenic lines. After 7-day stress, all the wild-type plants permanently died, while the transgenic lines could present recovery of vegetative growth and reproductive growth partially after being rewatered for 14 days (Figure 4(a)). Without salt treatment, the SOD activities of CK and transgenic lines were 159.5, 158.5, 154.1, and 167.5 U/g*·*FW, respectively, and there was no significant difference between them. Following salt stress treatment for 5 d, the SOD activities were 225.4, 298.9, 327.0, and 270.2 U/g*·*FW. At all durations of salt stress, the SOD activity in* CCHA-2142*-overexpressing plants was significantly higher than that in control plants (Figure 4(b)).

Lipid peroxidation is a marker of oxidative damage under high salinity, and we measured MDA content as an indicator of lipid peroxidation (Figure 4(c)). Without salt stress treatment, the MDA content of the control and transgenic plants was similar. With salt stress treatment of 1, 3, and 5 d, the MDA content changed by only a small amount in transgenic plants but increased dramatically in control plants. These results indicate that* CCHA-2142* overexpression led to reduced lipid peroxidation and thus likely increased tolerance to salt stress.

The membrane integrity of leaves was assessed by measuring electrical conductivity, which reveals the degree of ion leakage from cellular membranes. Although electrical conductivity increased in both transgenic and control plants in response to salt treatment, the degree of ion leakage increased less in the transgenic lines at 3 and 5 d than in the control plants at those time points (Figure 4(d)). This suggests that overexpressing* CCHA-2142* helps to maintain membrane permeability under high salt conditions, directly or indirectly.

Without salt stress, no significant differences in the contents of total chlorophyll, chlorophyll a, and chlorophyll b were detected between the control and* CCHA-2142*-overexpressing plants. However, after salt stress for 1 d, 3 d, and 5 d, the total chlorophyll content was reduced more dramatically in the control than in the transgenic plants (Figure 4(e)). In addition, the chlorophyll a content and chlorophyll b content have the same solution (Figures 4(f) and 4(g)). These results suggest that the photosynthetic capacity of the transgenic plants overexpressing* CCHA-2142* remained higher during salt treatment than that of the control plants.

## 4. Discussion

The cellular mechanisms of stress tolerance are similar from bacteria to higher plants, and halotolerant fungi are good potential sources of biotechnologically important genes. As abiotic stress could affect the cellular gene-expression machinery, it is evident that a lot of genes are up- or downregulated. For instance, heterologous expression of selected* Debaryomyces hansenii* and* Hortaea werneckii* genes markedly improved the salt tolerance of* Saccharomyces cerevisiae* [25, 26]. Lots of cellular responses to salt stress are conserved between the prokaryotes and the eukaryotes. Due to the similarity of the general cellular stress responses, these significant genes related to salinity tolerance have been studied by their overexpression in simple organisms, such as bacteria or yeast [7, 27].

*A. glaucus* (strain CCHA), which thrives on salt mines, represents another rich resource to mine for genes involved in salt tolerance. To isolate genes of potential biotechnological utility, we constructed a full-length cDNA library from* A. glaucus* under salt stress. Given that some of the protective mechanisms are common to both prokaryotes and eukaryotes under stress conditions [10, 28], we expressed the* A. glaucus* cDNA library in the model prokaryote* E. coli*, taking advantage of its short life cycle and ease of manipulation. The identification of over 100 transformants with increased halotolerance demonstrates the efficiency of this screening system for identification of candidate genes involved in basic cellular stress responses.

Characterization and identification of genes in halotolerant or halophilic microorganisms provide an important basis to elucidate the mechanisms underlying stress tolerance [29]. About 60% of the candidate genes identified in this study had not been characterized previously, and we confirmed their potential roles in salt stress using spot assays. All five clones selected for more detailed analysis and their putative functions were identified by comparison with GenBank database entries. PsRPL30E (ribosomal protein-L30E), one of the components of the large (60 S) ribosomal subunit from* Pisum sativum*, confers high salinity resistance to* E. coli*, and the expression of* PsRPL30E* is induced by high salt stress and repressed by cold stress in (*Pisum sativum*) pea [3]. The ribosomal structure between eukaryotes and prokaryotes is different; however, it may confer tolerance via a pathway yet to be illuminated. A yeast ribosomal 60 S subunit homologue was induced more than 2.0-fold under salt stress [30]. Here, we found that* CCHA*-*2247*, encoding a ribosomal 60 S subunit homologue, improved the salinity tolerance of the* E. coli*. This, along with its expression profile, suggests that* CCHA-2247* may be involved in salinity stress tolerance and thus may represent a new salt-tolerance candidate gene from* A. glaucus* (CCHA).

Another* A. glaucus* gene conferring salt tolerance to* E. coli*,* CCHA*-*2229*, encodes a protein that appears to be a member of the COGO428 superfamily, which includes divalent heavy-metal cations transporters playing an important role in zinc homeostasis at the cellular level [31]. The low affinity system has a lower affinity for substrate, and it is temperature, time, and concentration dependent. In* Saccharomyces cerevisiae*, the* ZRT2* gene encodes the low affinity zinc transporter which could increase low affinity uptake [32]. Considering CCHA-2229 with 8 transmembrane domains, the gene works as an ion transporter when suffering from abiotic stress. In addition,* CCHA-2114*, which was induced more than 3.2-fold under salt stress in this study, belongs to the DJ-1/Pfpl family. The* Saccharomyces cerevisiae HSP31* gene, also a member of the DJ-1/Pfpl family, protects cells against oxidative stress and complements other stress protection systems within the cell [24]. Although no similar sequences were found in the database,* CCHA-2221* also improved the salinity tolerance of* E. coli* cells.* CCHA-2221* was induced more than 2.57-fold under salt stress, supporting the idea that it plays a role in halotolerance. Finally, we found that to be induced more than 4.4-fold under salt stress,* CCHA-2142* encodes a protein containing a DUF3431 domain.

In order to assess the function of these candidate halotolerance genes,* CCHA-2142* was chosen as a representative gene to express in* Arabidopsis thaliana*. As is known to all, chlorophyll content, MDA content, and the degree of membrane damage are important indicators of injury and aging [33, 34]. Under salt stress, plant cells produce scavengers (mainly SOD) to remove free radicals induced by environmental stress. SOD can eliminate the accumulation of reactive oxygen species to reduce the damage to cellular membrane structures [35]. Abiotic stress can decrease chlorophyll content by inhibiting chloroplast formation and biosynthesis rates [36]. In our study, the levels of SOD in* CCHA-2142*-expressing plants under salt stress were higher than those in control plants, suggesting that* CCHA-2142* overexpression may enhance the tolerance of transgenic plants through influencing the levels of reactive oxygen scavengers. The* CCHA-2142*-expressing plants appeared to suffer less stress in the presence of salt, as evidenced by less reduction in chlorophyll content, less MDA accumulation, and less membrane damage compared to control plants. These data further confirmed and highlighted the functions of* A. glaucus CCHA-2142* in enhancing plant salt tolerance. However, the functional mechanism of this gene still needs further research. In view of the constitutive CaMV 35S promoter is active in all tissues and growth phases, the stress-inducible promoter rd29A is considered in the further experiment. This kind of promoter could direct gene expression only in induced osmotic stress and minimize the negative effective effects on plant growth [37, 38]. Also as there may be codon preference in different species, it would be considerable to improve the genetic code of these target genes due to the codon usages in our donor and recipient strains.

In this work, we identified a number of candidate genes related to salt tolerance in* A. glaucus*, which may shed light on novel mechanism of salinity tolerance. Overall, this study provided an important contribution for our better understanding of stress tolerance and showed that intensified studies of this halophilic eukaryotic microorganism are warranted to promote understanding of the targets, processes, and networks involved in salt tolerance, to increase our understanding of fundamental stress responses, and to identify genes that might enhance the properties of plants. However, It remains to identify these salinity stress induced genes to reveal a clear pathway for further experimentation.

## 5. Conclusion

In our study, a cDNA library from* A. glaucus* under salt stress was constructed by random overexpression, and a lot of genes related to salt tolerance were functionally annotated. Subsequently, five putative clones were selected from* E. coli* cells using LB agar with 1 M NaCl. Meanwhile, the analysis of* CCHA-2142*-expression transgenic* Arabidopsis thaliana* confirmed this gene could increase salt stress tolerance. All these results showed the utility of this cDNA library as a tool for isolating genes related to salt tolerance.

## Supplementary Material

Bioinformatic analysis of A. glaucus CCHA-2247, CCHA-2229 CCHA-2142.

## Figures and Tables

**Table 1:** Primers used in this study.

Primer	Primer sequence	Description
SMART IV oligonucleotide	5′-AAGCAGTGGTATCAACGCAGAGTGGCCATTACGGCCGGG-3′	First strand synthesis, forward
CDS III/3′ PCR primer	5′-ATTCTAGAGGCCGAGGCGGCCGACATG-d(T)_30_N–1N-3′ (N = A, G, C, or T; N–1 = A, G, or C)	First strand synthesis, reverse
T7	5′-CGCACAATCCCACTATCCTTCGCAAG-3′	Transgenic screening primer, forward
PYES2G-R	5′-GATAACATCGCAAGACCGGCAACAGG-3′	Transgenic screening primer, reverse
CCHA-42-F	5′-CGGACGAAATCCAACCCTTC-3′	Real-time PCR primer, forward
CCHA-42-S	5′-TTACCCGCGCACTAAGGTCA-3′	Real-time PCR primer, reverse
CCHA-14-F	5′-CCGGGCAAAAGGAGCATAAA-3′	Real-time PCR primer, forward
CCHA-14-S	5′-AACCCTGCTCATGCTGGACA-3′	Real-time PCR primer, reverse
CCHA-29-F	5′-ATTTACGGCCGGGGATATGG-3′	Real-time PCR primer, forward
CCHA-29-S	5′-ACGGGCCCCATTACCGAATA-3′	Real-time PCR primer, reverse
CCHA-21-F	5′-GGGTGAATGCGACCHAAACA-3′	Real-time PCR primer, forward
CCHA-21-S	5′-CCAGCGCTCGATCGTCTTTT-3′	Real-time PCR primer, reverse
CCHA-47-F	5′-CTTGATGCCCCHACGTTACA-3′	Real-time PCR primer, forward
CCHA-47-S	5′-TGCGCGCTCAAATGACAAAT-3′	Real-time PCR primer, reverse
Actin2-F	5′-GCACCCTGTTCTTCTTACCG-3′	Standard control primer, forward
Actin2-R	5′-AACCCTCGTAGATTGGCACA-3′	Standard control primer, forward
Tubulin1-F	5′-GAGTTGACCCAGCAGATG-3′	Standard control primer, forward
Tubulin1-R	5′-CTGGTTCTTGTTCTGGAC-3′	Standard control primer, reverse

**Table 2:** *A. glaucus *coding sequences expressed during growth on 3 M NaCl. One hundred cDNA clones were randomly selected and sequenced.

*A. glaucus* unigenes	Annotation (based on similarity to database sequences)
0307, 23171	RRM superfamily, nuclear and cytoplasmic polyadenylated RNA-binding protein pub1

2229, 2243, 23119, and 0501	ZIP zinc/iron transport family, plasma membrane low affinity zinc ion transporter

0508, 2247, 2372, 2390, 0209, 2302, 2341, 2380, 2382, 23144, 23151, 23159, and 2378	Ribosomal_L27e superfamily, 60S ribosomal protein L27e

0509, 23136, and 2018	Similar to alpha/beta hydrolase fold-3 domain containing protein

0314	Six-hairpin glycosidase

1402, 2037, and 2113	4HTB_3 (beta-hydroxydecanoyl-acyl carrier protein-(ACP-) dehydratase) structure and subsequently in 4HBT (4-hydroxybenzoyl-CoA thioesterase)

1408, 23140, and 23134	RNase_PH superfamily, exosome complex endonuclease 2/ribosomal RNA processing protein

2250	MFS general substrate transporter (secondary transporters that include uniporters, symporters, and antiporters)

2330	Casein kinase II beta 2 subunit

2334	Putative intracellular protease/amidase

2354, 23179, 2042, 2048, 2142, and 2263	DUF3431 superfamily

2362	WD repeat protein (adaptor/regulatory modules in signal transduction, Pre-mRNA processing and cytoskeleton assembly; typically contains a GH dipeptide 11–24 residues)

23131	p450 superfamily, cytochrome P450 [secondary metabolites biosynthesis, transport, and catabolism/methylsterigmatocystin oxidoreductase]

23149	PF03619 domain protein (organic solute transporter Ostalpha)

23207	WD40 superfamily. Nuclear pore complex subunit (adaptor/regulatory modules in signal transduction)

2265	Spore germination lipase lipC

2251	Family 2 glycosyl transferase

2258	Phosphoprotein

0203	7 transmembrane receptors

2301	Exodeoxyribonuclease V subunit beta

2302, 2304	Protein CRE_17637

2305	Aspartic proteinase nepenthesin-1-like

2306	Carnitine acetyl transferase

2339	TIM_phosphate_binding superfamily, nucleoporin SONB

2377	PDI_b_family, PDI_a_family (thioredoxin_like superfamily), disulfide-isomerase precursor

2383, 23163	MDR superfamily, delta-aminolevulinic acid dehydratase

23103	PING superfamily, regulator of gluconeogenesis Rmd5

23104	Fasciclin superfamily, fasciclin domain family protein

23120	Lactamase_B superfamily, Zn-dependent hydrolases of the beta-lactamase fold

23135	NADH dehydrogenase subunit 1

23187	Rho_GDI superfamily (rho-gdp dissociation inhibitor)

23189	P-loop NTPase domain superfamily (ras-like GTP-binding protein)

2397	SocE

2253, 2227, 2214, 2242, 2369, 23156, 23197, 0215, 0217, and 2346	DJ-1/PfpI family protein (Type 1 glutamine amidotransferase- (GATase1-) like domain)

2252	Isonitrile hydratase

22651, 2375, 23102, 23143, 23160, 23168, 23191, and 2248	4-Methyl-5 (B-hydroxyethyl)thiazole monophosphate biosynthesis protein

2243	Zinc/iron permease

22652, 2389, and 2246	Unknown; no similar proteins found

**Table 3:** *A. glaucus *coding sequences that were identified from the cDNA expression library as putatively conferring increased salt-resistance.

*A. glaucus* unigenes	Annotation (based on similarity to database sequences)
2229	ZIP zinc/iron transport family, plasma membrane low affinity zinc ion transporter
2247	Ribosomal_L27e superfamily, 60S ribosomal protein L27e
1402	4HTB_3 (beta-hydroxydecanoyl-acyl carrier protein- (ACP-) dehydratase) structure and subsequently in 4HBT (4-hydroxybenzoyl-CoA thioesterase)
1408	RNase_PH superfamily, exosome complex endonuclease 2/ribosomal RNA processing protein
2227, 2114	DJ-1/PfpI family protein (Type 1 glutamine amidotransferase- (GATase1-) like domain)
2246, 22652, and 2221	Unknown; no similar proteins found
2250	MFS general substrate transporter (secondary transporters that include uniporters, symporters, and antiporters)
2258	Phosphoprotein
2142	DUF3431 superfamily
22651, 2248	4-Methyl-5 (B-hydroxyethyl)thiazole monophosphate biosynthesis protein
22654	Spore germination lipase lipC
0203	7 transmembrane receptors

**Table 4:** *A. glaucus *coding sequences that were obtained from the cDNA expression library selected with increased salt-resistance.

GenBank accession number	Gene	Putative gene function	Selection
KJ934998	CCHA-2142	DUF3431 superfamily	1 M NaCl
KJ935001	CCHA-2114	DJ-1/PfpI family protein (Type 1 glutamine amidotransferase- (GATase1-) like domain)	1 M NaCl
KJ934999	CCHA-2229	COGO428 superfamily (plasma membrane low affinity zinc ion transporter)	1 M NaCl
KJ935002	CCHA-2221	Unknown; no similar proteins found	1 M NaCl
KJ935000	CCHA-2247	60S ribosomal protein L27e	1 M NaCl

## References

[1] Margesin R., Schinner F. (2001). Potential of halotolerant and halophilic microorganisms for biotechnology. *Extremophiles*.

[2] Singh P., Thumar J. T., Gohel S. D., Purohit M. K. (2010). Molecular diversity and enzymatic potential of salt-tolerent alkaliphilic actinomycetes. *Current Research, Technology and Education Topics in Applied Microbiology and Microbial Biotechnology*.

[3] Joshi A., Dang H. Q., Vaid N., Tuteja N. (2009). Isolation of high salinity stress tolerant genes from pisum sativum by random overexpression in *Escherichia coli* and their functional validation. *Plant Signaling and Behavior*.

[4] Kültz D. (2003). Evolution of the cellular stress proteome: from monophyletic origin to ubiquitous function. *Journal of Experimental Biology*.

[5] Forment J., Naranjo M. Á., Roldán M., Serrano R., Vicente O. (2002). Expression of Arabidopsis SR-like splicing proteins confers salt tolerance to yeast and transgenic plants. *The Plant Journal*.

[6] Kanhonou R., Serrano R., Ros Palau R. (2001). A catalytic subunit of the sugar beet protein kinase CK2 is induced by salt stress and increases NaCl tolerance in Saccharomyces cerevisiae. *Plant Molecular Biology*.

[7] Rausell A., Kanhonou R., Yenush L., Serrano R., Ros R. (2003). The translation initiation factor elF1A is an important determinant in the tolerance to NaCl stress in yeast and plants. *Plant Journal*.

[8] Mundree S. G., Whittaker A., Thomson J. A., Farrant J. M. (2000). An aldose reductase homolog from the resurrection plant Xerophyta viscosa baker. *Planta*.

[9] Yamada A., Saitoh T., Mimura T., Ozeki Y. (2002). Expression of mangrove allene oxide cyclase enhances salt tolerance in *Escherichia coli*, yeast, and tobacco cells. *Plant and Cell Physiology*.

[10] Yamada A., Tsutsumi K., Tanimoto S., Ozeki Y. (2003). Plant RelA/SpoT homolog confers salt tolerance in *Escherichia coli* and *Saccharomyces cerevisiae*. *Plant & Cell Physiology*.

[11] Gostinčar C., Gunde-Cimerman N., Turk M. (2012). Genetic resources of extremotolerant fungi: a method for identification of genes conferring stress tolerance. *Bioresource Technology*.

[12] Jin Y., Weining S., Nevo E. (2005). A MAPK gene from Dead Sea fungus confers stress tolerance to lithium salt and freezing-thawing: prospects for saline agriculture. *Proceedings of the National Academy of Sciences of the United States of America*.

[13] Mai Z., Yang J., Tian X., Li J., Zhang S. (2013). Gene cloning and characterization of a novel salt-tolerant and glucose-enhanced *β*-glucosidase from a marine streptomycete. *Applied biochemistry and biotechnology*.

[14] Prista C., Soeiro A., Vesely P., Almagro A., Ramos J., Loureiro-Dias M. C. (2002). Genes from Debaryomyces hansenii increase salt tolerance in Saccharomyces cerevisiae W303. *FEMS Yeast Research*.

[15] Liu X., Liu J., Wei Y., Tian Y., Fan F., Pan H., Zhang S. (2011). Isolation, identification and biologic characteristics of an extreme Halotolerant *Aspergillus* sp. *Journal of Jilin University (Science Edition)*.

[16] Qiu W. M. (2012). *Construction of Screening System for Cd Stress-Tolerance Related Genes in Sedum Alfredii Hance*.

[17] Livak K. J., Schmittgen T. D. (2001). Analysis of relative gene expression data using real-time quantitative PCR and the 2(-Delta Delta C(T)) Method. *Methods*.

[18] Yang L., Wei J., Dai C., Zhu Y. M. (2008). To establish osmotic stress related gene system by high-throughput screening technology. *China Biotechnology*.

[19] Clough S. J., Bent A. F. (1998). Floral dip: a simplified method for Agrobacterium-mediated transformation of Arabidopsis thaliana. *Plant Journal*.

[20] Giannopolitis C. N., Ries S. K. (1977). Superoxide dismutases: I. Occurrence in higher plants. *Plant Physiology*.

[21] Heath R. L., Packer L. (1968). Photoperoxidation in isolated chloroplasts. I. Kinetics and stoichiometry of fatty acid peroxidation. *Archives of Biochemistry and Biophysics*.

[22] Arnon D. I. (1949). Copper enzymes in isolated chloroplasts. Polyphenoloxidase in beta vulgaris. *Plant Physiology*.

[23] Chen C., Deutscher M. P. (2005). Elevation of RNase R in response to multiple stress conditions. *The Journal of Biological Chemistry*.

[24] Skoneczna A., Miciałkiewicz A., Skoneczny M. (2007). Saccharomyces cerevisiae Hsp31p, a stress response protein conferring protection against reactive oxygen species. *Free Radical Biology and Medicine*.

[25] Prista C., González-Hernández J. C., Ramos J., Loureiro-Dias M. C. (2007). Cloning and characterization of two K+ transporters of Debaryomyces hansenii. *Microbiology*.

[26] Prista C., Loureiro-Dias M. C., Montiel V., García R., Ramos J. (2005). Mechanisms underlying the halotolerant way of Debaryomyces hansenii. *FEMS Yeast Research*.

[27] Soto A., Allona I., Collada C., Guevara M.-A., Casado R., Rodriguez-Cerezo E., Aragoncillo C., Gomez L. (1999). Heterologous expression of a plant small heat-shock protein enhances Escherichia coli viability under heat and cold stress. *Plant Physiology*.

[28] Liu Y., Zheng Y. (2005). PM2, a group 3 LEA protein from soybean, and its 22-mer repeating region confer salt tolerance in Escherichia coli. *Biochemical and Biophysical Research Communications*.

[29] Yadav N. S., Rashmi D., Singh D., Agarwal P. K., Jha B. (2012). A novel salt-inducible gene SbSI-1 from Salicornia brachiata confers salt and desiccation tolerance in E. coli. *Molecular Biology Reports*.

[30] Yale J., Bohnert H. J. (2001). Transcript expression in Saccharomyces cerevisiae at high salinity. *Journal of Biological Chemistry*.

[31] Gaither L. A., Eide D. J. (2001). Eukaryotic zinc transporters and their regulation. *BioMetals*.

[32] Zhao H., Eide D. (1996). The ZRT2 gene encodes the low affinity zinc transporter in *Saccharomyces cerevisiae*. *Journal of Biological Chemistry*.

[33] Shrestha A., Grantz D. A. (2005). Ozone impacts on competition between tomato and yellow nutsedge. *Crop Science*.

[34] Zhang S., Li N., Gao F., Yang A., Zhang J. (2010). Over-expression of *TsCBF1* gene confers improved drought tolerance in transgenic maize. *Molecular Breeding*.

[35] Sairam R. K., Tyagi A. (2004). Physiology and molecular biology of salinity stress tolerance in plants. *Current Science*.

[36] Sharma P., Sharma N., Deswal R. (2005). The molecular biology of the low-temperature response in plants. *BioEssays*.

[37] Yamaguchi-Shinozaki K., Shinozaki K. (1993). Arabidopsis DNA encoding two desiccation-responsive rd29 genes.. *Plant Physiology*.

[38] Pellegrineschi A., Reynolds M., Pacheco M., Brito R. M., Almeraya R., Yamaguchi-Shinozaki K., Hoisington D. (2004). Stress-induced expression in wheat of the *Arabidopsis thaliana* DREB1A gene delays water stress symptoms under greenhouse conditions. *Genome*.

