# Supplementary material for: Isolation of Salt Stress-Related Genes from Aspergillus glaucus CCHA by Random Overexpression in Escherichia coli
Source: ScientificWorldJournal. 2014 Oct 14;2014:620959. doi: 10.1155/2014/620959 (PMC4212599; doi:10.1155/2014/620959)

Isolation of salt stress-related genes from *Aspergillus glaucus* CCHA by random overexpression in *Escherichia coli*^1^

Jie Fang^a,b*^, Xiaojiao Han^a,b*^, Lihua Xie^a,b*^, Mingying Liu^a,b^, Guirong Qiao^a,b^, Jing Jiang^a,b^, and Renying Zhuo^a,b2^

Author for correspondence:

Renying Zhuo

Tel: 86-571-63311860

Email: zhuory@gmail.com

Fig. S1 Bioinformatic analysis of *A. glaucus* *CCHA-2247*, CCHA-*2229 CCHA-2142*. 2. (a) Alignment of the deduced amino acid sequence of the *CCHA-2247* with *Byssochlamys spectabilis* No. 5 (GAD94676.1), *Aspergillus clavatus* NRRL 1(XP_001269541.1), *Aspergillus flavus* NRRL3357 (XP_002381915.1), *Aspergillus terreus* NIH2624 (XP_001212185.1), *Neosartorya fischeri* NRRL 181 (XP_001265033.1), *Penicillium digitatum* Pd1 (EKV08347.1) and *Talaromyces marneffei* ATCC 18224 (XP_002143603.1). (b) Phylogenetic tree of *A. glaucus* CCHA-2247 and *Aspergillus fumigatus* Af293 (XP_750383), *Aspergillus niger* CBS 513.88(XP_001398956), *Aspergillus flavus* NRRL3357 (XP_002381915), *Aspergillus oryzae* RIB40 (BAE56933), *Penicillium digitatum* PHI26 (EKV09886), *Schizosaccharomyces pombe* 972h-] (NP_596141), *Pyrenophora tritici-repentis* Pt-1C-BFP (XP_001932225), *Arabidopsis thaliana* (AEC08651), and *Medicago truncatula* (AES70144). (c) Alignment of the deduced amino acid sequence of *CCHA-2229* with *Aspergillus clavatus* NRRL 1 (XP_001272799.1), *Aspergillus fumigatus* Af293 (XP_749518.1), *Aspergillus ruber* CBS 135680 (EYE97956.1), *Aspergillus oryzae* RIB40 (XP_001818429.1), *Penicillium chrysogenum* Wisconsin 54-1255 (XP_002562334.1), *Aspergillus niger* CBS 513.88 (XP_001389092.1) and *Aspergillus niger* CBS 513.88 (XP_001389092.1). (d) Phylogenetic tree of *A. glaucus CCHA-2229* and *Aspergillus fumigatus* A1163 (EDP54043)*, Aspergillus fumigatus* (AAT11931), *Aspergillus clavatus* NRRL 1 (XP_001272799), *Aspergillus terreus* NIH2624 (XP_001218120), *Aspergillus oryzae* RIB40 (XP_001818429), *Aspergillus nidulans* FGSC A4 (XP_661403), *Macrophomina phaseolina* MS6 (EKG15420), *Candida dubliniensis* CD36 (XP_002418215), and *Wickerhamomyces ciferrii* (CCH44363)*.* (e) Alignment of the deduced amino acid sequence of *CCHA-2142* with *Aspergillus clavatus* NRRL 1 (XP_001274931.1), *Aspergillus flavus* NRRL3357 (XP_002382575.1), *Aspergillus fumigatus* Af293 (XP_746495.1), *Aspergillus nidulans* FGSC A4 (XP_660077.1)*, Aspergillus terreus* NIH2624 (XP_001213981.1) *and Neosartorya fischeri* NRRL 181 (XP_001262501.1). (f) Phylogenetic tree of *A. glaucus CCHA-*2142 and *Aspergillus flavus* NRRL3357 (XP_002382575), *Aspergillus oryzae* RIB40 (BAE61337), *Aspergillus terreus* NIH2624 (XP_001213981), *Neosartorya fischeri* NRRL 181(XP_001262501), *Aspergillus kawachii* IFO 4308 (GAA87067), *Aspergillus niger* ATCC 1015 (EHA27709), *Aspergillus nidulans* FGSC A4 (XP_660077), *Arthroderma gypseum* CBS 118893 (XP_003174795), and *Trichophyton rubrum* CBS 118892 (XP_003233680)


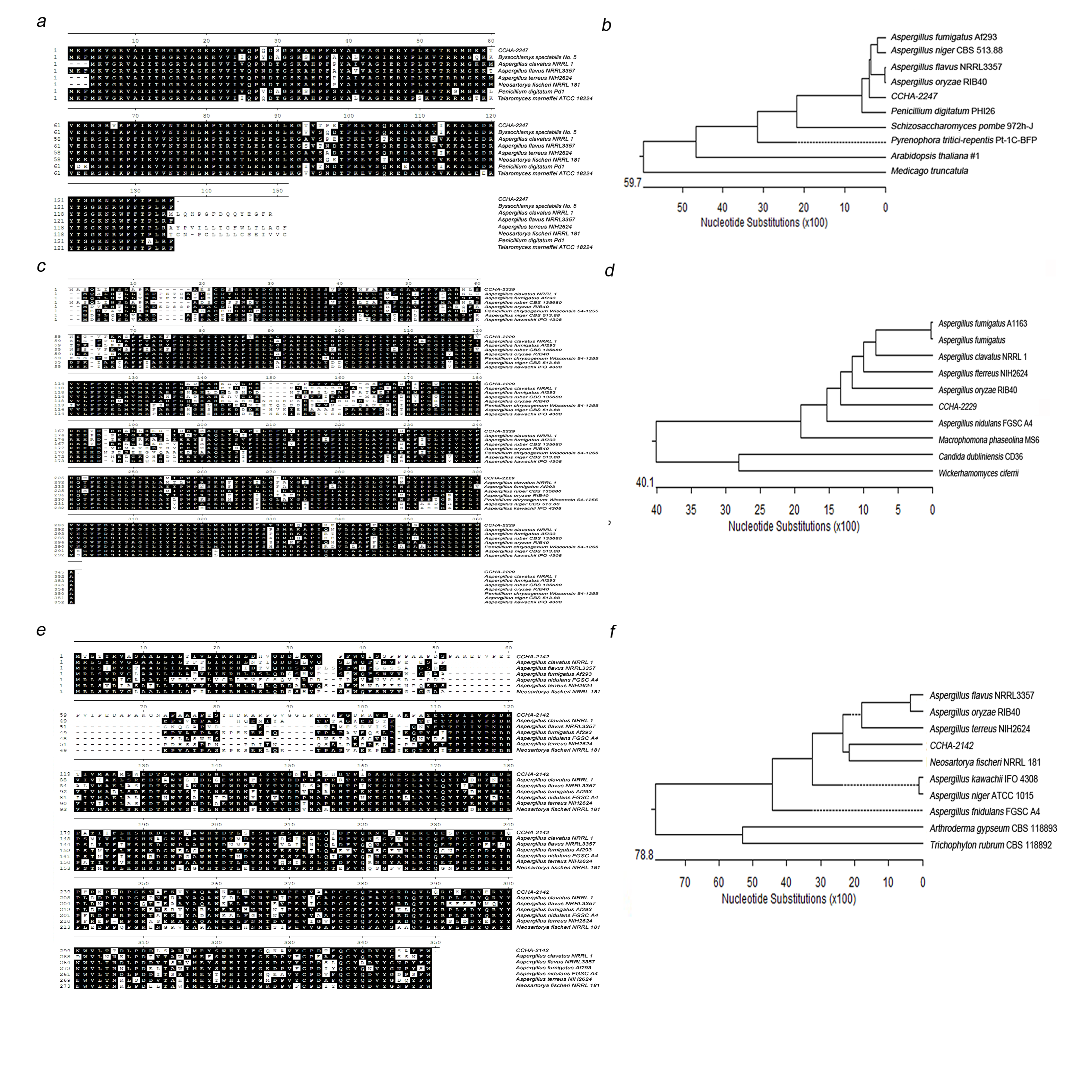

Supplement: Supplementary file 1 — Bioinformatic analysis of A. glaucus CCHA-2247, CCHA-2229 CCHA-2142. [file 620959.f1.doc]
